# Extracorporeal membrane oxygenation-supported one-stop surgery for transcatheter aortic valve replacement and percutaneous coronary intervention in high-risk complex coronary artery and aortic valvular disease: a case report

**DOI:** 10.3389/fcvm.2025.1604387

**Published:** 2025-09-10

**Authors:** Zhihan Zhang, Dongsheng Wang, Yanan Yu, Beibei Du, Zhongfan Zhang, Guohui Liu

**Affiliations:** Department of Cardiology, China-Japan Union Hospital of Jilin University, Changchun, Jilin, China

**Keywords:** transcatheter aortic valve replacement, extracorporeal membrane oxygenation, aortic stenosis, percutaneous coronary interventions, mechanical circulatory support, case report

## Abstract

Aortic stenosis (AS) is frequently associated with coronary artery disease (CAD), particularly in high-risk patients for whom treatment strategies lack standardized clinical guidelines. We report a case of a patient with severe AS and high-risk CAD, leading to significant heart failure, for whom surgical aortic valve replacement and coronary artery bypass grafting were unsuitable because of very high risk of morbidity and mortality. The patient underwent a one-stop procedure combining extracorporeal membrane oxygenation (ECMO)-assisted transcatheter aortic valve replacement (TAVR) and percutaneous coronary intervention (PCI). During the procedure, the patient first received preventive veno-arterial ECMO placement, successfully underwent PCI on the right coronary artery and left anterior descending artery, and then TAVR was performed without complications. The patient tolerated the procedure well, with hemodynamics remaining stable throughout. At one-year follow-up, the patient's heart function was significantly improved. This case provides valuable experience in treating high-risk AS combined with CAD, demonstrating the feasibility and effectiveness of this approach in clinical practice.

## 1 Introduction

Aortic stenosis (AS) is a condition characterized by the narrowing of the aortic valve orifice, which impedes blood flow into the systemic circulation. Due to the high overlap of pathophysiology and risk factors, a significant number of patients with AS also have coronary artery disease (CAD) (1). The widespread use of catheter-based interventional therapies has made this patient population increasingly challenging to overlook. Patients with both AS and CAD generally require a comprehensive treatment approach addressing both diseases. While current guidelines provide some recommendations for managing patients with severe AS complicated by CAD, clear treatment strategies remain lacking for high-risk patients with severe heart failure and a high risk of circulatory collapse. Consequently, this report presents a case of a high-risk CAD patient with severe AS and severely impaired cardiac function who underwent a one-stop surgical treatment combining extracorporeal membrane oxygenation (ECMO)-assisted transcatheter aortic valve replacement (TAVR) and percutaneous coronary intervention (PCI).

## 2 Case presentation

**Figure 1:**
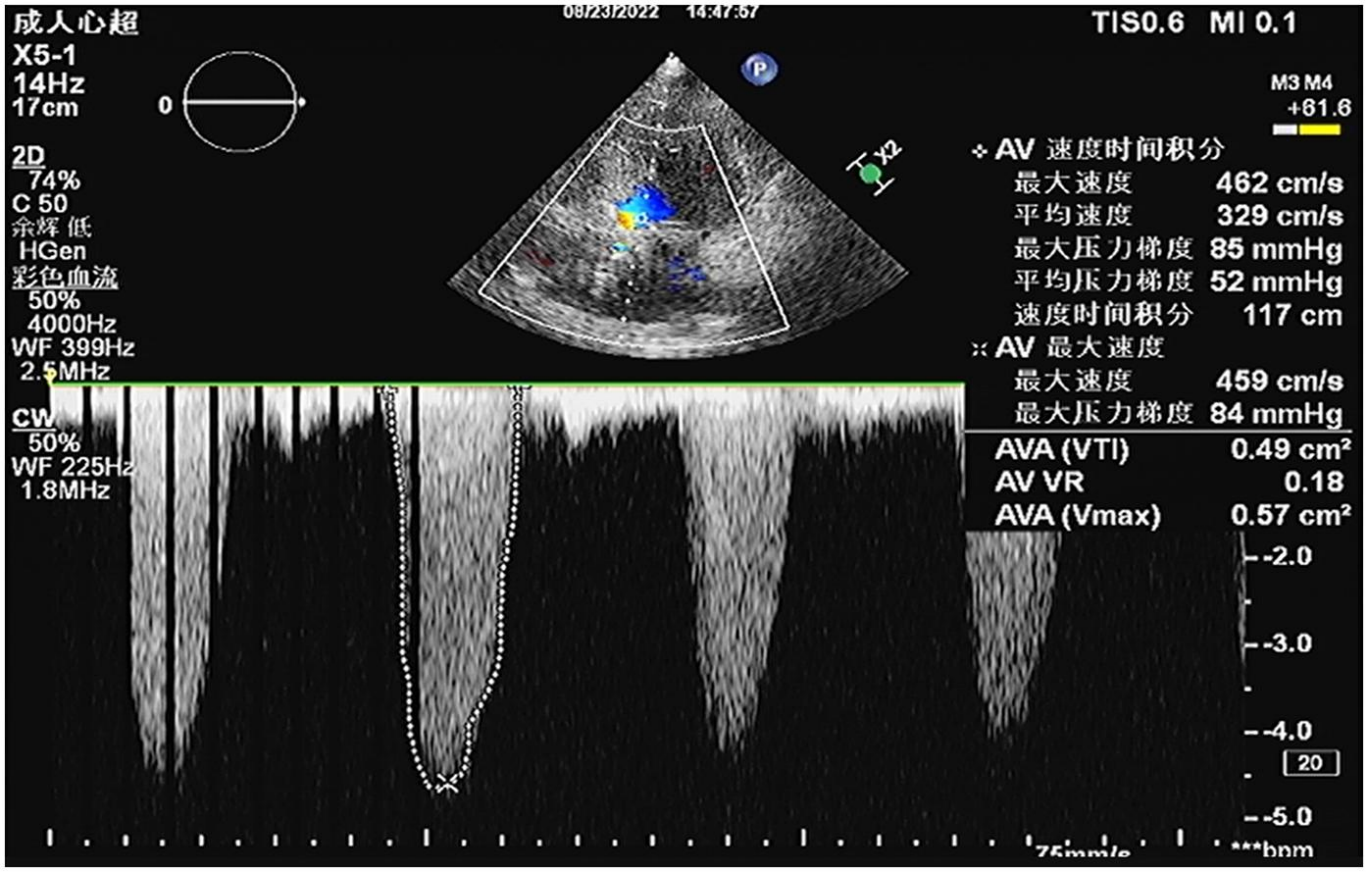
Preoperative transthoracic echocardiography (TTE) revealed severe aortic valve stenosis with a peak transvalvular velocity of 4.6 m/s, peak pressure gradient (PG) of 85 mmHg, mean pressure gradient (mPG) of 52 mmHg, aortic valve area (AVA) by velocity-time integral (VTI) of 0.49 cm^2^, and aortic regurgitant area of 4.6 cm^2^.

A 64-year-old female patient presented with persistent shortness of breath for two days, which was aggravated by mild exertion, and an inability to lie flat at night. Her medical history included hypertension and a cerebrovascular accident (stroke) for over 10 years. Upon admission, the patient was alert, and physical examination revealed a systolic ejection murmur at the aortic valve area, a systolic blowing murmur at the mitral valve area, and moist rales in both lungs. A 12-lead electrocardiogram indicated sinus rhythm, with a Q wave in lead III, T-wave inversion in leads I and aVL, and ST-segment elevation of approximately 0.1 mV in leads V1–V4. Leads V5 and V6 exhibited flat T-waves. Laboratory tests revealed troponin I level of 2.400 ng/ml (reference: 0.010–0.023 ng/ml) and N-terminal pro-B-type natriuretic peptide levels of 2030.00 ng/L (reference: 300.00–900.00 ng/L). Transthoracic echocardiography (TTE) demonstrated severe AS with moderate aortic regurgitation, a peak aortic valve flow velocity of 4.6 m/s, and a mean transvalvular pressure gradient of 52 mmHg (Figure 1). Severe mitral regurgitation was noted, with a regurgitant area of 10 cm^2^. There was generalized left ventricular hypokinesis, and the left ventricular ejection fraction (LVEF) was 36.6%.

**Figure 2:**
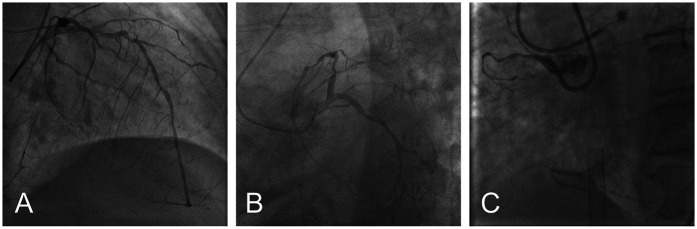
Preoperative coronary angiography: severe ostial stenosis of LAD **(A)** severe proximal stenosis of LCX **(B)** total occlusion of RCA **(C)** (LAD, left anterior descending artery; LCX, left circumflex artery; RCA, right coronary artery).

Based on these findings, the patient was diagnosed with severe AS, acute myocardial infarction, Killip III, and acute heart failure. Subsequently, the patient was provided with non-invasive positive pressure ventilation and pharmacologic treatment to correct heart function, significantly relieving her dyspnea. Coronary angiography (CAG) revealed 90% stenosis with calcification in the proximal left anterior descending artery (LAD), 70%–80% stenosis in the mid-segment, with thrombolysis in myocardial infarction (TIMI) flow grade 3, and retrograde perfusion to the right coronary artery (RCA) via the LAD (Figure 2A). The proximal left circumflex artery indicated 50%–60% stenosis, with TIMI flow grade 3 (Figure 2B), and RCA was completely occluded (Figure 2C). During CAG, the patient developed acute left heart failure and was promptly transferred to the coronary care unit (CCU) for further management of heart failure. A multidisciplinary consultation was held to discuss whether to proceed with open-heart surgery or interventional treatment and the choice of anesthetic approach. Given the patient's critical condition, with severe AS, complex CAD, and low ejection fraction, the risks of surgical aortic valve replacement (SAVR) and coronary artery bypass grafting (CABG) were considered extremely high. Our center has extensive clinical experience with ECMO-assisted TAVR in treating severe heart failure. Considering the patient's specific condition, the team determined that a combination of ECMO-assisted PCI and TAVR would be the safest approach for treatment. To reduce the risk of cardiac arrest, the treatment plan involved initial ECMO insertion under light sedation, followed by endotracheal intubation. After thoroughly informing the patient's family about the risks and treatment plan, the family signed the informed consent form. On the 10th day of hospitalization, the patient underwent the procedure in the interventional operating room.

**Figure 3:**
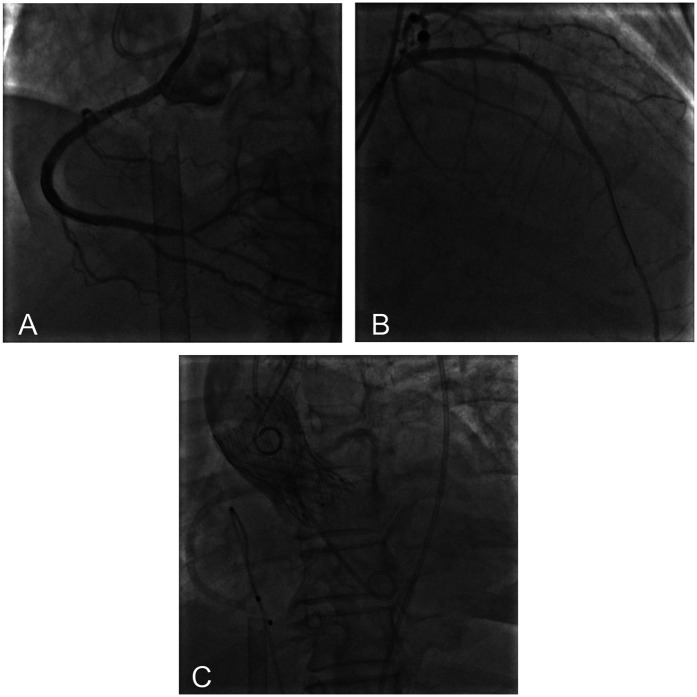
Postoperative imaging showed successful recanalization of RCA **(A)** and LAD **(B)** aortic root angiography showed optimal valve positioning with patent coronary ostia **(C).**

Under light sedation with dexmedetomidine, veno-arterial (V-A) ECMO (Maquet, Getinge AB, Sweden) was successfully inserted using the left femoral venous and arterial access using 21Fr venous cannula and 17Fr arterial cannula. Under dual CAG guidance with a 7F Amplatz Left (AL)1.0 guiding catheter (right femoral access) (Beijing Demax Medical Technology Co., Ltd., China) and a 6F Extra Back-up (EBU)3.5 guiding catheter (right radial access)(Medtronic, Inc.,USA), a Runthrough guidewire (Terumo Co., Japan) was advanced to the RCA acute marginal branch. An ultrasound catheter was used to locate the opening of the occluded segment of the RCA. XT-R guidewire (Asahi Intecc Co., Japan) under the support of Finecross microcatheter (Terumo Co., Japan) successfully recanalized the occluded RCA. Retrograde angiography confirmed the guidewire in the true lumen. After sequential pre-dilation using a 1.5 × 12 mm balloon (Boston Scientific Co., USA), 2.0 × 20 mm balloon (Yinyi Biotech, China), and 2.25 × 13 mm scoring balloon (Goodman Co., Ltd, Japan) at 8–12 atm. Three stents were deployed from distal to proximal: A 2.5 × 38 mm stent (Boston Scientific Co., USA) and two 3.0 × 30 mm stents [Essen Technology (Beijing) Co., Ltd.] (Figure 3A). Afterwards, the stenotic lesion in the LAD was dilated and stented with a 2.75 × 38 mm stent (Boston Scientific Co., USA) and a 3.0 × 25 mm stent [Essen Technology (Beijing) Co., Ltd.] (Figure 3B).

**Figure 4:**
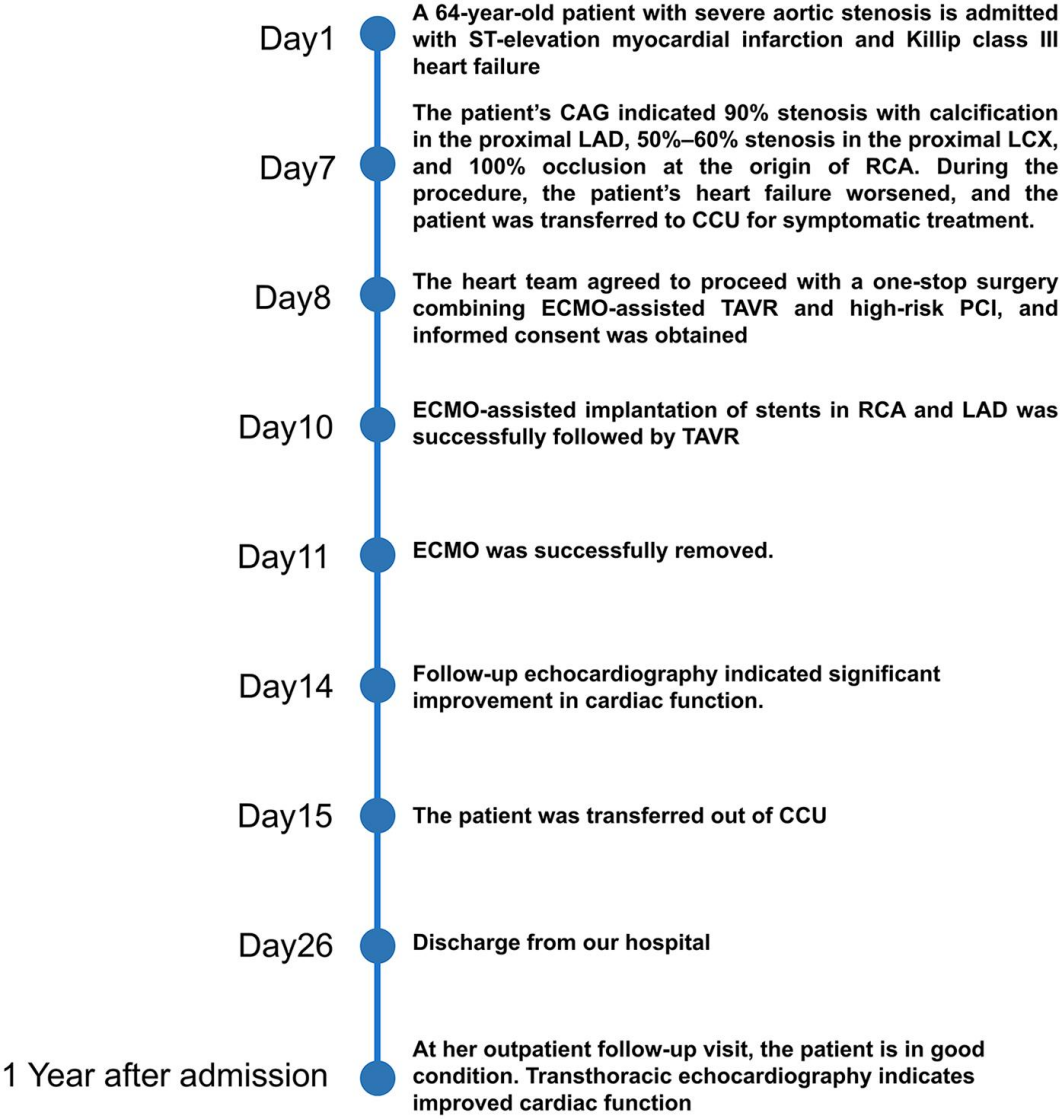
Timeline of treatment.

A floating electrode (St. Jude Medical, USA) was placed using the right femoral vein into the right ventricular apex, and a 6F pig-tail catheter was introduced through the right radial artery into the non-coronary sinus. Angiography revealed a calcified aortic valve with moderate regurgitation. The right femoral artery served as the primary access route for the TAVR procedure, while the right radial artery served as the secondary access route. The AL1.0 angiographic catheter and a 260 cm straight guidewire (Terumo Corporation, Japan) successfully passed through the aortic valve into the left ventricle, with the catheter advanced to the apex. A pig-tail catheter was exchanged to measure the left ventricular to aortic root pressure gradient, which was 50 mmHg. The pre-shaped 260 cm COOK stiff guidewire (Cook Medical, USA) was delivered to the apex, and balloon dilation was performed using a 20 mm × 4cm × 100 cm balloon at the aortic valve area under rapid pacing at 180 beats per minute. The QiMing heart valve delivery system (Hangzhou QiMing Medical Devices Co., Ltd, China) was exchanged to the aortic valve root, and the valve release position was confirmed under angiographic guidance. After the slow release of the 23 mm aortic valve under 180 beats per minute pacing, a final angiogram was performed to verify optimal positioning before removing the pig-tail catheter and fully releasing the aortic valve. The valve delivery system was then withdrawn. Post-procedural angiography at the aortic root indicated minimal aortic regurgitation, no coronary involvement, and slight contrast leakage around the aortic valve (Figure 3C). Repeat measurement of the left ventricular to aortic root pressure gradient indicated a reduction to 0 mmHg. During the procedure, TTE confirmed optimal valve positioning with minimal aortic regurgitation. The patient's vital signs were stable postoperatively, and she was successfully transferred back to the CCU. On the second postoperative day, the patient's dyspnea significantly improved, and ECMO was successfully weaned. The left femoral arterial access was closed using the Perclose ProGlide suture-mediated closure system (Abbott Vascular, USA), while the left femoral venous access was secured with the figure-of-eight suture technique. At one-year follow-up, TTE revealed a marked improvement in LVEF, from 36.6% to 72.3%. A non-significant increase in aortic valve regurgitation was observed (PVR area increased from 2.2 cm^2^ at postoperative 2 months to 2.6 cm^2^ at 1 year), and the mitral regurgitation area decreased from 10 to 1.5 cm^2^ (Table 1). Figure 4 summarized the patient's treatment history by way of a timeline.

**Table 1:** Changes in echocardiographic parameters.

Echocardiographic parameter	Time point				
	Preoperative	Postoperative Day 1	Postoperative Day 4	Postoperative 2 Months	Postoperative 1 Year
AV Vmax (m/s)	4.6	2.2	2.2	2.37	2.4
AV media PG (mmHg)	52	19	11	14	12
AV max PG (mmHg)	85	–	20	22	23
LVEF (%)	36.6	39	52.6	59.3	72.3
MR Area (cm^2^)	10	6.8	6.5	2.7	1.5
PVR Area (cm^2^)	–	6.7	2.9	2.2	2.6

AV Vmax, aortic maximum velocity; AV media PG, aortic mean gradient; AV max PG, aortic peak gradient; LVEF, left ventricular ejection fraction; MR Area, mitral regurgitation area; PVR Area, paravalvular regurgitant area post-TAVR.

## 3 Discussion

For patients with AS combined with CAD, selecting the appropriate treatment options and clinical decision-making present significant challenges. Several international guidelines and clinical consensus have provided treatment recommendations for this complex patient population in recent years. However, specific treatment strategies still pose challenges and remain controversial.

The 2018 ESC guidelines on myocardial revascularization and the 2021 ESC guidelines on managing valvular heart disease recommend that PCI be considered for patients undergoing TAVR if the proximal coronary artery diameter stenosis exceeds 70% (2, 3). The 2023 ESC clinical consensus statement has not significantly changed this recommendation for such patients (4). However, these guidelines and statements primarily target patients with stable CAD, and there remains a lack of clear treatment guidance for high-risk patients with complex coronary lesions or acute heart failure. The 2023 ESC scientific statement on acute heart failure and valvular heart disease highlights that there is insufficient clinical evidence regarding whether and when to perform PCI in acute heart failure patients undergoing TAVR. Consequently, complex patient treatment decisions rely on close collaboration within the heart team and individualized judgment (5). The 2020 ACC/AHA valvular heart disease guidelines suggest that for AS patients with severe CAD, particularly those with complex bifurcation left main or multi-vessel disease and a SYNTAX score greater than 33, combined SAVR and CABG is superior to TAVI with PCI (6). However, our patient had poor general status (frailty, heart failure, multiple comorbidities), with an ASA classification of up to class IV, making her unable to tolerate anesthesia and having a high perioperative mortality risk.

In this case, the patient presented with severe AS, and CAG revealed multi-vessel CAD, with a LVEF of 36%, besides the patient has typical exertional angina (Canadian Cardiovascular Society Class IV) meeting the criteria for Complex Higher Risk Indicated Percutaneous Coronary Intervention (CHIP) patient. Currently, no clear clinical guidelines provide a unified treatment protocol for such patients; consequently, a multidisciplinary consultation was organized. After discussion, the surgical team determined that the anesthesia and surgical risks were extremely high and decided against traditional open-heart surgery. In this context, a combined approach of TAVR and PCI became the necessary intervention. Mechanical circulatory support was considered essential for ensuring the patient's safety, given the high risk of hemodynamic instability or even cardiac arrest during the procedure. To thoroughly evaluate and select the most appropriate mechanical circulatory support device, we compared the characteristics of Impella and V-A ECMO in TAVR procedures. Impella, as a local mechanical circulatory support device, primarily enhances ventricular pumping function to increase cardiac output, thereby improving systemic perfusion and coronary blood flow (7). However, its support capacity in TAVR is limited: the Impella catheter may interfere with the valve frame, affecting the precise deployment of the valve. Case reports have indicated that collisions between the Impella catheter and TAVR implants can lead to valve displacement or damage (8–10). Additionally, the FDA previously issued a large-scale recall of the device due to incidents where the rotating impeller broke after colliding with TAVR implants, with the resulting fragments potentially entering the bloodstream, causing embolism and increasing the risk of stroke or organ ischemia. In contrast, V-A ECMO provides systemic circulatory support, effectively maintaining systemic perfusion and oxygenation. This facilitates better management of hemodynamic instability that may occur during combined PCI and TAVR procedures while also delivering reliable respiratory support (11). Furthermore, V-A ECMO technology is well-established, with related case reports and small-scale studies demonstrating that prophylactic use of V-A ECMO in high-risk patients yields favorable outcomes (12–15). In this case, selecting V-A ECMO as the support method during the procedure was considered the more appropriate decision. Whether to perform complex interventional procedures simultaneously or in stages remains debatable, even with left ventricular support. Performing them may result in worse outcomes, primarily due to the extended surgical duration and the increased use of contrast agents, which can negatively impact prognosis (16). In this case, the patient's right coronary chronic total occlusion PCI was successfully performed with antegrade recanalization using intravascular ultrasound guidance. The procedure involved minimal contrast agent use and a relatively short duration, which the patient tolerated well. Consequently, TAVR was completed during the same procedure.

Regarding the treatment sequence, we chose to perform PCI first, followed by TAVR, to avoid obstructing the coronary ostium with the prosthetic valve and prevent interference with the coronary intervention. Moreover, addressing the coronary artery stenosis first facilitates better myocardial tolerance during TAVR, particularly when rapid pacing is used during the procedure, which may otherwise induce myocardial ischemia.

Currently, there are limited reports on the treatment strategies for high-risk patients with severe AS and CAD, particularly regarding the prophylactic use of VA-ECMO for perioperative circulatory support and the concurrent treatment of TAVR and PCI. The approach used in this case represents a highly innovative strategy. Through close collaboration within the heart team, meticulously designed surgical steps, and timely circulatory support, we completed the one-stop procedure combining PCI and TAVR, providing valuable reference experience for the treatment of similar cases in the future.

## Data Availability

The raw data supporting the conclusions of this article will be made available by the authors, without undue reservation.
